# sMICA as novel and early predictors for acute myocardial infarction

**DOI:** 10.1186/s40001-016-0220-2

**Published:** 2016-06-16

**Authors:** Cunyu Fu, Yunxiang Shi, Zongqin Yao

**Affiliations:** Department of Cardiovascular Medicine, Yishui Central Hospital, Linyi, Shandong China

**Keywords:** Acute myocardial infarction, MHC class I polypeptide-related chain A, Troponin T

## Abstract

**Background and aims:**

MHC class I polypeptide-related chain A (MICA) molecule is induced in response to viral infection, various types of stress, such as endoplasmic reticulum stress, and ischemia or/and reperfusion, by which MICA was shed from the cell surface into the extracellular domain, generating a soluble form (sMICA). In the present study, we designed to investigate the serum sMICA level in patients with AMI and determine whether sMICA could be an early biomarker for diagnosis of AMI.

**Methods:**

There were 103 patients who presented with first-time AMI that was assessed after the incident. The control group consisted of 103 healthy volunteers. Serum levels of sMICA and Troponin T were detected by the specific ELISA kits.

**Results:**

Serum levels of sMICA reach the peaks [(1.34 ± .18 and 1.72 ± .20)n/l] at 6–12 h and serum levels of cTnT reach the peaks [(1.16 ± .28 and 1.14 ± .34)n/l] at 12–24 h. Both of them were significantly higher than the healthy controls [(.168 ± .014) n/l, p = .000] for sMICA and [(.13 ± .06) n/l, p = .000] for Troponin T (cTnT). sMICA is more sensitive in the early diagnosis of AMI than cTnT. The combined ROC analysis revealed an AUC value of .78 (95 % CI .69–.83) in discriminating AMI patients from healthy controls.

**Conclusions:**

We have detected high levels of sMICA in patients with AMI. Elevated serum sMICA may be a novel biomarker for the early detection of myocardial injury in humans.

## Background

Coronary artery disease (CAD) and acute myocardial infarction (AMI) are the leading causes of death in developed and developing countries [1]. AMI accounts for most of the mortality due to CAD. Cardiac myoglobin and creatine kinase-MB are used as biomarkers for diagnosing AMI [2], and plasma cTnI is widely used in clinical practice as the gold standard for diagnosing AMI [3]. However, elevated plasma cTnI was not only observed in certain cardiac ischemic injury, but also in some other diseases, such as severe heart failure, atrial fibrillation, chronic kidney disease, severe sepsis, septic shock, etc. [4–6]. Circulating biomarkers of myocardial damage, especially cardiac specific troponin, have facilitated the early diagnosis of AMI, maximizing the benefits of revascularization therapy. In AMI patients, troponin levels increase as early as 6 h (h) after the onset of chest pain. However, due to the relative delay in the timing of the release of troponin, it is necessary to find novel and effective biomarkers for the early and accurate diagnosis of AMI [7].

The non-classical major histocompatibility complex class I (MHCI) molecule A (MICA) has a molecular structure similar to that of the classical MHCI molecules [8]. MICA is a natural ligand for the activating receptor natural killer group 2, member D (NKG2D) expressed on the surface of natural killer (NK) cells. The binding of MICA to NKG2D triggers a cascade of signal transduction events that activate NK cells to release cytotoxic molecules and, subsequently, cause NK cells to identify and lyse target cells. Many malignant carcinoma cells express high levels of MICA on their surface, making them susceptible to targeting and killing by NK cells [9].

Under the physiologic conditions, MICA is only expressed in epithelial cells of the gastrointestinal tract and is only present at very low levels in most normal cells and tissues. However, it is activated by a variety of stimuli, such as endoplasmic reticulum stress, and ischemia or/and reperfusion [10–12]. Previous studies suggest that in addition to expressing membrane bound MICA, stress-inducible tissues MICA also have a mechanism to shed MICA from the cell surface into the extracellular domain, generating a soluble form (sMICA) [10].

In the present study, we investigate whether sMICA was elevated in patients with AMI. Then, we assess the clinical significance of sMICA level in patients with AMI. This is the first time in literature to investigate the clinical significance of sMICA in AMI.

## Methods

### Ethics statement

Experiments were conducted according to the Declaration of Helsinki. This study was supported and approved by the Ethics Committee of Yishui Central Hospital. All participants formally consented to participate in all stages of the study.

### Study population

Patients >18 years of age presenting to the emergency department with symptoms suggestive of AMI with an onset or peak within the last 24 h were recruited after providing written informed consent. 103 patients with AIM were enrolled. The clinical characteristics of all patients are given in Table 1. All the AMI patients were diagnosed according to the international criteria. Patients with previous MI or percutaneous coronary intervention (PCI), any chronic peritoneal or hemodialysis, acute or chronic infection, significant hepatic dysfunction, kidney failure (glomerular filtration rate (GFR) < 15 mL/min/1.73 m^2^ or on dialysis), or known or treated malignancies were excluded. 103 healthy adult volunteers (normal ECG and no history of cardiovascular diseases, the same exclusion criteria) were enrolled as controls. All controls, who had the same exclusion criteria and no history of any cardiovascular disease, came from the *Medical Examination Center* of the Yishui Central Hospital of Linyi. Full blood count and routine biochemistry indices were determined invenous blood. Creatine kinase-MB (CK-MB) and cardiac specific troponin T (TnT) were measured in serum immediately after arrival at the hospital as markers of myocardial damage.

**Table 1:** Baseline characteristics of the patients

Characteristic	Control	AMI	p value
Age (years)	60 ± 12.2	61.3 ± 11.4	.54
Sex: male/female	55/48	60/43	.36
BMI (kg/m^2^)	23.6 ± 4.1	24.7 ± 4.3	.518
Waist (cm)	93.4 ± 12.4	94.2 ± 13.1	.83
SBP (mm Hg)	119.3 ± 21.4	132.4 ± 22.7	.428
Body mass index (kg/m^2^)	67.5 ± 13.3	68.8 ± 13.6	.46
SBP (mm Hg)	119.3 ± 21.4	132.4 ± 22.7	.236
DBP (mm Hg)	76.3 ± 12.2	88.4 ± 11.5	.614
WBC (x10^3/^ul)	6.36 ± 1.23	11.15 ± 2.17	<.001
HB (g/dl)	13.1 ± 1.56	14.4 ± 1.42	.184
TG (mmol/L)	1.32 ± .75	1.53 ± .84	.165
Cholesterol (mg/dL)	166.5 ± 32.5	168.7 ± 34.4	.546
FBS (mg/dL)	86.5 ± 15.4	97.2 ± 14.6	.176
HDL (mg/dL)	46.8 ± 13.4	41.5 ± 11.0	.342
CK-MB (U/L)	18.27 ± 7.43	46.1 ± 42.3	.001
Cardiac troponin T (ng/l)	.13 ± .06	1.31 ± .14	.000
sMICA (ng/l)	.128 ± .014	1.72 ± .23	.000

### Routine clinical assessment

Complete blood count, including white blood cell (WBC), hemoglobin (Hb), and chemical profiles, including blood urea nitrogen (BUN), creatinine (Cr), uric acid, total cholesterol (TC), triglyceride (TG), high-density lipoprotein cholesterol (HDL), and low-density lipoprotein cholesterol (LDL) were checked by venous blood sampling. The initial cardiac enzymes, including CK-MB and cardiac specific troponin T (cTnT), were measured on just after arrival at the hospital as markers of myocardial damage using venous blood. The CK-MB was checked every 8 h. The cTnT was then checked as the time of chest pain depicted in Table 2.

**Table 2:** Circulating cTnT and sMICA levels (ng/l) in AMI

Time of chest pain	Time of arrival at the hospital									
	0 h		3 h		6 h		12 h		24 h	
	cTnT	sMICA	cTnT	sMICA	cTnT	sMICA	cTnT	sMICA	cTnT	sMICA
0–1 h (n = 4)	.15 ± .07	.19 ± .13	.26 ± .05	1.14 ± .02^#^	.62 ± .07	1.72 ± .67^#^	1.36 ± .28^*^	1.24 ± .18^#^	1.24 ± .34^*^	.46 ± .024
1–2 h (n = 7)	.18 ± .12	.34 ± .08	.31 ± .06	1.63 ± .25^#^	.61 ± .12^*^	1.52 ± .17^#^	1.14 ± .26^*^	.78 ± .07	1.12 ± .29^*^	.212 ± .04
2–3 h (n = 10)	.21 ± .07	.84 ± .09	.52 ± .15^*^	1.71 ± .06^#^	.89 ± .32^*^	1.17 ± .06^#^	1.25 ± .27^*^	.6 ± .05	1.08 ± .26^*^	.197 ± .048
3–4 h (n = 23)	.28 ± .06	1.012 ± .11^#^	.57 ± .13^*^	1.42 ± .11^#^	1.08 ± .27^*^	.92 ± .07^#^	1.33 ± .32^*^	.56 ± .05	1.16 ± .28^*^	.173 ± .048
4–5 h (n = 20)	.36 ± .09	1.528 ± .1^#^	.75 ± .23^*^	1.22 ± .14^#^	1.13 ± .24^*^	.71 ± .07	1.25 ± .26^*^	.292 ± .04	1.04 ± .21^*^	.18 ± .04
5–6 h (n = 16)	.53 ± .12^*^	1.64 ± .16^#^	.92 ± .17^*^	1.02 ± .16^#^	1.17 ± .26^*^	.57 ± .065	1.19 ± .27^*^	.27 ± .032	1.12 ± .23^*^	.199 ± .04
6–12 h (n = 19)	.94 ± .35^*^	1.20 ± .10^#^	1.13 ± .25^*^	.68 ± .09	1.09 ± .23^*^	.376 ± .09	1.06 ± .24^*^	.279 ± .04	.96 ± .21^*^	.19 ± .04
12–24 h (n = 4)	1.26 ± 1.16^*^	.386 ± .08	1.16 ± .14^*^	.255 ± .05	1.15 ± .18^*^	.24 ± .05	1.07 ± .20^*^	.29 ± .05	.97 ± .23^*^	.18 ± .05

Vs control value: ^*^ *p* = .000; ^#^ *p* = .000

### Serum sMICA measurements

Initial serum sMICA was measured on just after arrival at the hospital, and then sMICA was checked as the time of chest pain depicted in Table 1. Briefly, 5 mL venous blood samples of patients with AMI and controls were collected in EDTA anticoagulant tubes at admission. Samples were centrifuged at 3000×*g* for 10 min at 4 °C, and then the supernatant was isolated and collected. Serum sMICA levels were measured using a commercially available kit (Human sMICA ELISA Kit). The intra-assay precision, expressed as coefficients of variation, was 4.6–8.4 %; the inter-assay precision was 5.3–8.6 %, and the sensitivity was <7.4 ng/l. All assays were performed in duplicate.

### Statistical analysis

Statistical treatment was performed using the SPSS 17.0 software (Chicago, IL, USA). Continuous variables were compared with the use of the Mann–Whitney-test and t test, as appropriate, and categorical variables with the use of the Pearson’s Chi-square test. Receiver operating characteristic (ROC) curves were constructed to assess the sensitivity and specificity of sMICA measurements obtained to compare its ability to diagnose AMI. Multiple logistic regression analysis was carried out for evaluating the combined diagnostic accuracy of circulating sMICA. All hypothesis testing was two-tailed, and P values of less than .05 were considered to indicate statistical significance without adjustments for multiple testing.

## Results

### Circulating CK-MB,Troponin T, and sMICA levels in AMI patients

We detected the circulating Troponin T (cTnT) value in AMI. The mean value was [1.31 ± .14] ng/l, which was significantly higher than the controls [(.13 ± .06) ng/l] (p = .000).Circulating CK-MB and sMICA levels in AMI patients was about [(46.1 ± 42.3)U/L] and [(1.72 ± .23] ng/l], which was significantly higher than the controls [(18.27 ± 7.43) U/L] (p < .01) and [(.128 ± .014] ng/l] (p = .000). Among 103 patients with AMI, the basic clinical characteristics of the patients in this study are shown in Table 1.

### sMICA is more sensitive than cTnT in the early diagnosis of AMI

We divided the AMI patients into several groups according to the time of chest pain in Table 2. Circulating cTnT and TGF-β1 values was detected at 0, 0–3, 3–6, 6–12, and 12–24 h after arrival at the hospital. Because the circulating CK-MB is less sensitive than cTnT, so we did not detected it for further investigation. As shown in Table 2, circulating cTnT was significantly enhanced when suffered from chest pain for 3–6 h and reached the peak levels at 12–24 h. However, circulating sMICA was significantly enhanced when suffered from chest pain for 3 h, and reached the peak levels at 6–12 h, then declined rapidly from 12 h, and restored to the normal levels at 24 h. Although cTnT was enhanced when suffered from chest pain for 0–3 h (.26 ± .05), there is not significantly different compared to the control (.12 ± .08) (p > .05). However, sMICA value was significantly enhanced when suffered from chest pain for 0–3 h (.937 ± .11), there is significantly different compared to the control (.172 ± .02) (p < .05). We, therefore, suggested that sMICA is more sensitive in the early diagnosis of AMI.

### Circulating sMICA levels as predictors of AMI

**Fig. 1:**
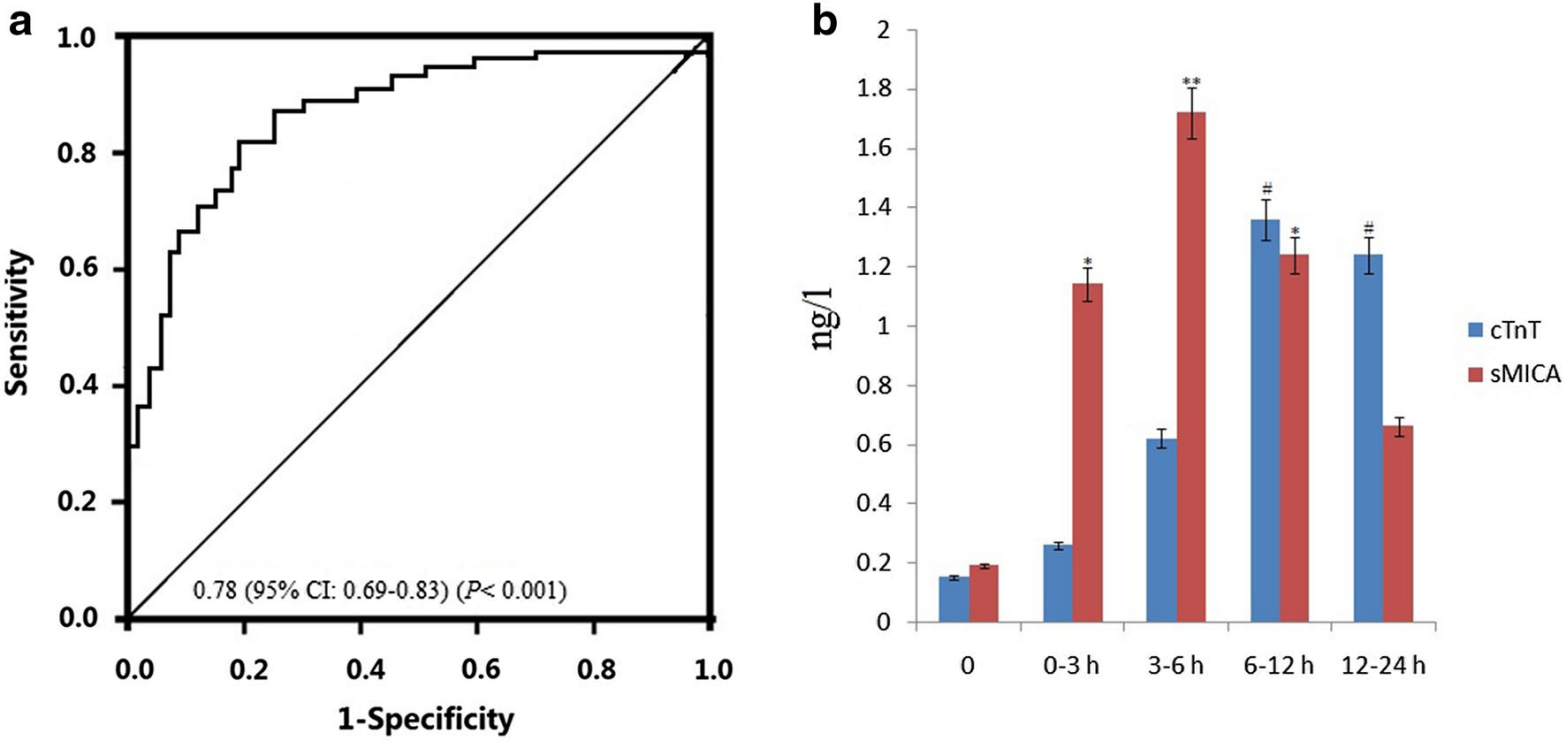
Effect of serum sMICA level in diagnosis of AMI. **a** Evaluation of serum sMICA for the diagnosis of AMI by the ROC curve analysis. **b** sMICA levels at presentation in relation to the time, since the onset of symptoms among patients found to have an acute myocardial infarction vs control, ^*^ *p* < .01; ^**^ *p* = .000; ^#^ *p* = .000

Figure 1a shows analysis according to the timing of the blood sample from outset on symptoms. TGF-β1 levels peaked at 3–6 h and TnT at 12–24 h. To further evaluate the predictive power of serum sMICA for AMI, ROC curve and areas under ROC curve (AUC) analyses were performed. As shown in Fig. 1b, the AUC of sMICA in AMI patients was .78 (95 % CI .69–.83) (p < .001). ROC curve analysis of sMICA exhibited strong differentiation power between AMI patients and healthy controls during the early phase of AMI.

## Discussion

Previous studies have found that MICA shedding is considered a principal mechanism of tumor cells to escape from NKG2D-mediated immunosurveillance in humans. MICA shedding not only results in a reduction of MICA surface density on tumor cells but also generates sMICA, which was shown to systemically down-regulate NKG2D on cytotoxic effector cells and to promote expansion of immunosuppressive, intratumoral CD4+ NKG2D+ T cells [13]. Recently, it has been found that MICA could be activated in the normal tissues by a variety of stimuli, such as endoplasmic reticulum stress, γ-irradiation, commonly used chemotherapeutic drugs, and ischemia/reperfusion-induced tissue injury [10–12]. The activated MICA could also shed to the MICA, and generate sMICA [11]. However, the function of sMICA generated in the stressed tissues is not clear.

Smith et al. [14] have reported that 15.8 % of cardiac allograft recipients produced sMICA, sMICA do not adversely affect the outcome of cardiac transplantation. However, Kauke et al. have found that sMICA may be related to adverse outcome after heart transplantation. Post-transplantation monitoring of sMICA antibodies could identify patients with an increased risk for acute rejection and vasculopathy [15]. Zou et al. have found that presensitization of kidney-transplant recipients against MICA antigens is associated with an increased frequency of graft loss and might contribute to allograft loss among recipients who are well matched for HLA [16].

In this study, serum sMICA was visibly increased in 103 AMI patients. The ROC analyses showed that the sMICA might be suitable diagnostic markers of AMI. In addition, we found that sMICA levels was significantly increased at 3 h, and peak at 12 h, and returned to the normal level at 24 h. However, Troponin levels increased at 6 h, peak at 12 h, and stay elevated after 24 h. We, therefore, suggested that sMICA could be an early marker for the diagnosis for AMI.

Although sMICA is activated in AMI, the function and mechanisms of sMICA are poorly understood. Experimental studies suggest that increased sMICA or MICA was an indicator of poor prognosis in cancer diseases [17–19]. Therefore, understanding the signaling mechanisms responsible for sMICA-mediated effects is important to design novel therapeutic strategies, targeting the sMICA signaling cascade in the infarcted and remodeling heart.

## Conclusion

In conclusion, this study has shown elevated sMICA concentrations in patients with AMI. Elevated sMICA in plasma may be a novel biomarker for the early detection of myocardial injury in humans. However, a large-scale prospective cohort study is necessary to determine the potential casual relationship between sMICA and AMI.
